# Clinical Role of Codon 87 of the CYFIP2 Gene in Early Infantile Epileptic Encephalopathy: A Clinical Case Description

**DOI:** 10.7759/cureus.35323

**Published:** 2023-02-22

**Authors:** Juliana Da Silva Cardoso, Rita Gomes, Maria Abreu, João Parente Freixo, Cáudia Falcão Reis, Cristina Garrido

**Affiliations:** 1 Pediatrics, Centro Materno Infantil do Norte Albino Aroso, Centro Hospitalar Universitário do Porto, Porto, PRT; 2 Genetics, Centro de Genética Médica Dr. Jacinto Magalhães, Centro Hospitalar Universitário do Porto, Porto, PRT; 3 Genetics, Center for Predictive and Preventive Genetics, Institute for Cell and Molecular Biology, University of Porto, Porto, PRT; 4 Pediatric Neurology, Centro Materno Infantil do Norte Albino Aroso, Centro Hospitalar Universitário do Porto, Porto, PRT

**Keywords:** next-generation sequencing (ngs), infant, early infantile epileptic encephalopathy, cyfip2 gene, codon 87

## Abstract

The diagnosis of early infantile epileptic encephalopathy (EIEE) remains challenging, and next-generation sequencing (NGS) techniques have played a key role in identifying genetic causes. Recent studies have shown an association between mutations in the *CYFIP2* gene and EIEE, with 20 deleterious variants reported so far and a *de novo* mutational hotspot at codon 87.

A male infant presented with seizures since the age of four months as well as significant developmental delay and microcephaly. The seizures were of different types, frequent and refractory to treatment, including different anticonvulsant drugs. Metabolic studies showed no significant changes. The initial electroencephalogram revealed bilateral paroxysmal activity with hemispherical diffusion. Brain MRI showed no pathological changes. Analysis of a whole exome sequencing (WES) based multigene panel for epilepsy disclosed a heterozygous *CYFIP2* gene variant [*c.258_266del; p.(Trp86_Ser88del)*] established as *de novo*.

We describe the case of an infant with EIEE due to a *de novo* heterozygous in-frame deletion of three amino acids in *CYFIP2*: c.258_266del; p.(Trp86_Ser88del). This in-frame deletion eliminates codon 87, a mutational hotspot associated with a particularly severe EIEE phenotype. All previous reports had missense variants with a presumably gain-of-function mechanism. The clinical picture of our patient is very similar to the ones with deleterious variants affecting codon 87 reported in the literature. Our case report is the first to describe a disease-causing in-frame deletion in *CYFIP2* and reiterates a consistent genotype-phenotype correlation.

## Introduction

The diagnosis of early infantile epileptic encephalopathy (EIEE) remains challenging, and next-generation sequencing (NGS) techniques have played a key role in identifying monogenic causes. Recent studies have shown an association between mutations in the *CYFIP2* gene and EIEE. *CYFIP* (cytoplasmic Fragile X Mental Retardation Protein, FMRP interacting protein) is a conserved protein family that is highly expressed at the neuronal synapse. It includes *CYFIP1* and *CYFIP2*, which share 88% homology. Both proteins play an essential role in FMRP function; however, they have different expression patterns and regulation mechanisms in the brain development process: *CYFIP*1 and FMRP are directly bonded and modulate the affinity between FMRP and mRNAs; *CYFIP2* has a process similar to *CYFIP1* and is still regulated by FMRP through *CYFIP2* mRNA translation [1].

In 2018, Nakashima M et al. identified three *de novo *missense variants at the Arg87 residue [c.259C>T (p.Arg87Cys), c.260G>T (p.Arg87Leu) and c.260G>C (p.Arg87Pro)] that caused gain-of-function effects on the Wiskott-Aldrich syndrome protein family (WAVE) signaling pathway in four unrelated individuals with EIEE. All individuals had intractable seizures (since they were six months) and severe psychomotor development delay. Other common features included microcephaly, facial dysmorphisms, and hypotonia. Initial interictal electroencephalograms showed a burst-suppression pattern and/or hypsarrhythmia, which were compatible with Ohtahara syndrome and West syndrome, respectively. Brain MRI revealed diffuse cerebral atrophy in three individuals, mainly in the frontal lobes [2].

Begemann A et al. described a fourth *de novo* missense variant at the Arg87 residue, c.259C>A (p.Arg87Ser), that was present in a patient with EIEE, global developmental delay, and microcephaly [3].

In 2019, Zweier M et al. reported 12 patients harboring eight *de novo CYFIP2* variants, seven different missense, and one splice site variant. These had a broader clinical spectrum, ranging from mild to moderate cognitive impairment without epilepsy to a similarly severe phenotype, including intractable epilepsy and profound intellectual disability [4].

The patient’s clinical information was retrospectively reviewed from the electronic medical files. Both the epilepsy NGS gene panel and parental genetic studies were performed with parental consent in an external laboratory in a clinical support setting, using Twist Human Core Exome Kit through the Illumina platform and PCR/Sanger sequencing. A literature review was carried out. Informed consent for publication was obtained from parents.

## Case presentation

**Figure 1:**
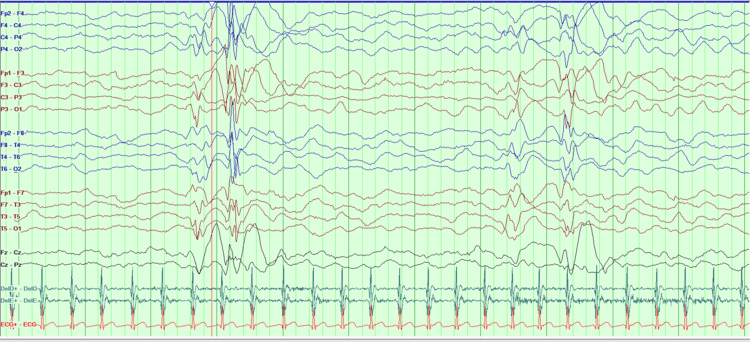
First EEG (0.1 sec, 30 Hz, S 10 uv). EEG: Electroencephalogram.

We report a case of a male born from nonconsanguineous healthy parents at 39 weeks of gestation and delivered by cesarean section. The pregnancy was uneventful. Ultrasounds and serological screening were normal. The neonatal APGAR scores at 1, 5, and 10 minutes were 8, 10, and 10, respectively. Birth anthropometry was appropriate for gestational age. Physical examination at birth was described as normal. He had a healthy brother and no relevant family history. He was admitted to the hospital twice (at the age of one and two months old) due to periods of irritability and feeding difficulties. The first episode was interpreted in the context of probable sepsis, and the latter was associated with a suspected allergy to cow's milk protein due to vomiting and feeding difficulties. Seizures started at four months with upward eye deviation, cyanosis, cervical hyperextension, and generalized hypertonia, followed by clonic movements of the four limbs that lasted seconds. Later, these seizures alternated with spasms and focal seizures with right eye deviation that increased in frequency over time. He also had alternated periods of drowsiness and unexplained irritability. By then, he presented peculiar facies (high and narrow forehead, bulbous upper lip) and significant neurologic impairment, being incapable of gaze fixation or pursuit, presenting marked axial hypotonia, poor cephalic control, poor suction reflex, and swallowing with tongue protrusion; he had hyperreflexia and Rossolimo reflex. The electroencephalogram (EEG) registered bilateral paroxysmal activity, particularly in the temporal regions and posterior quadrants, with hemispherical diffusion. Background rhythm was poorly defined, and sleep spindles were undeveloped (Figure 1).

**Figure 2:**
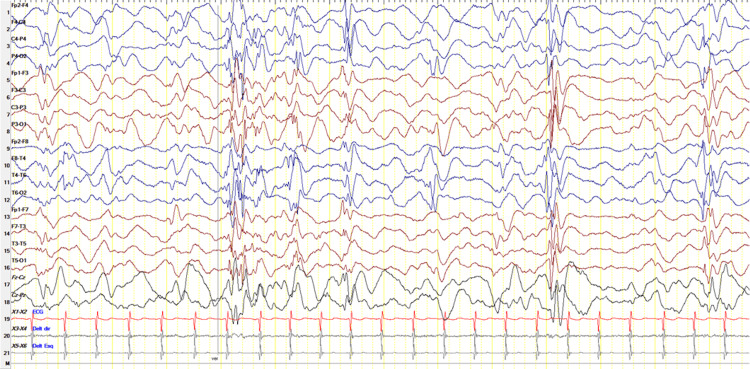
Electroencephalographic evolution of the patient (0.1 sec 30 Hz, S 10 uv).

He was started on therapy with levetiracetam without any improvement. Phenobarbital was later associated with mild benefits. When he started having infantile spasms, and there was evidence of small fragments of disorganized activity on EEG, vigabatrin was also started with transitory clinical improvement. In order to try to achieve better clinical control, a ketogenic diet was implemented, and there was partial clinical control of seizures for several months, despite maintaining scarce interaction and marked hypotonia. He was hospitalized at 6, 7, and 10 months due to febrile episodes associated with worsening epileptic seizures. Since he was ten months, his epilepsy aggravated, and he started having several small myoclonic, focal, and tonic seizures and spasms daily. Zonisamide was tried, but no clinical benefit was evident. EEG showed numerous bursts of abrupt waves, spikes, and slow delta waves from 1 to 2.5-3 Hz, bihemispheric, with posterior predominance. They occurred in bursts but more often as "discharges" of about one second in duration, followed by attenuation or near suppression of the signal. Associated with bursts-suppressions, beta rhythms were very sporadically observed at 14-16 Hz, more frontocentral (spindles) (Figure 2). He maintained refractory epilepsy despite being medicated with vigabatrin, phenobarbital, valproic acid, and a ketogenic diet.

At the most recent evaluation (16 months old), he had microcephaly, severe neurodevelopmental delay (global hypotonia, spontaneous eye opening without gaze fixation or pursuit, and incoordinate spontaneous movements), was fed exclusively by nasogastric tube, and was under mechanical ventilatory support during sleep.

An extensive work-up was performed, including biochemicals and metabolic studies and a 3T brain MRI that showed no relevant changes. Analysis of a whole exome sequencing-based multigene panel for epilepsy disclosed a heterozygous in-frame deletion of three amino acids in CYFIP2: NM_001291722.1 c.258_266del; p.(Trp86_Ser88del), classified by the laboratory as a variant of unknown significance (VUS). Segregation study through sequencing in the parents revealed the variant was *de novo*, and activation of the PM6 criteria allowed its reclassification as likely pathogenic.

## Discussion

We describe the case of an infant with EIEE due to a *de novo *heterozygous in-frame deletion of three amino acids in *CYFIP2*: c.258_266del; p.(Trp86_Ser88del). This in-frame deletion eliminates codons 86 to 88. Codon 87 is a mutational hotspot where missense variants cause a particularly severe EIEE phenotype, as previously reported in the literature. Nakashima M et al. presented three missense variants at the Arg87 residue, all with a clinical phenotype of early-onset refractory epilepsy and severe developmental delay [2]. The clinical picture of our patient was very similar to that described by Nakashima M et al., which supports a consistent genotype-phenotype correlation caused by modification of Arg87 and a pivotal role of this residue in protein stabilization.

However, the initial clinical presentation of our patient overlaps with many other cases of EIEE, and an extensive etiological study will always have to be carried out since there are no specific characteristics directing clinicians toward targeted investigations.

The prevalence of *de novo* variants in *CYFIP2* implicated in EIEE is not yet reported. However, previous studies have evaluated the occurrence of *de novo *mutations in patients with EIEE, including identifying mutations in *CYFIP2*. Peng J et al. performed WES in 56 Chinese families with West syndrome and reported an average number of *de novo* variants in each family of 0.41 (including identification of *de novo* mutations in the *CYFIP2*) [1]. Considering the scarcity of information recurrence of *de novo* variants in *CYFIP2* and the possibility of gonadal mosaicism, genetic counseling is challenging and fundamentally based on a theoretic recurrence risk due to gonadal mosaicism, which has not yet been described for this gene.

In the literature so far, the prognosis of children with these new variants has been described as poor since they fail to respond to the currently available anti-seizure medication and usually display severe developmental delays. Aside from specific genetic counseling, an advantage of identifying a genetic cause raises the possibility of developing targeted gene therapies that correct or modify the error. However, in this regard, precision therapy is still in the nascent stage and lacks credible clinical application [5].

## Conclusions

We describe the case of an infant with EIEE due to a *de novo* heterozygous in-frame deletion of three amino acids in *CYFIP2*: c.258_266del; p.(Trp86_Ser88del). This in-frame deletion eliminates codon 87, a mutational hotspot associated with a particularly severe EIEE phenotype. The clinical picture of our patient is very similar to the ones with deleterious variants affecting codon 87 reported in the literature. Our case report is the first to describe a disease-causing in-frame deletion in *CYFIP2* and reiterates a consistent genotype-phenotype correlation.
